# Targeted Therapies in Cholangiocarcinoma: Emerging Evidence from Clinical Trials

**DOI:** 10.3390/medicina55020042

**Published:** 2019-02-08

**Authors:** Maria Maddalena Simile, Paola Bagella, Gianpaolo Vidili, Angela Spanu, Roberto Manetti, Maria Antonietta Seddaiu, Sergio Babudieri, Giordano Madeddu, Pier Andrea Serra, Matteo Altana, Panagiotis Paliogiannis

**Affiliations:** Department of Medical, Surgical, and Experimental Sciences, University of Sassari, Viale San Pietro 43, 07100 Sassari, Italy; simile@uniss.it (M.M.S.); paola.bagella@tiscali.it (P.B.); gianpaolovidili@uniss.it (G.V.); angela.spanu@email.it (A.S.); rmanetti@uniss.it (R.M.); maseddaiu@uniss.it (M.A.S.); babuder@uniss.it (S.B.); giordano@uniss.it (G.M.); paserra@uniss.it (P.A.S.); matteoaltana@alice.it (M.A.)

**Keywords:** cancer, biliary tree, cholangiocarcinoma, molecular pathways, genomics, targeted therapies

## Abstract

Cholangiocarcinoma (CCA) is a highly-aggressive malignancy arising from the biliary tree, characterized by a steady increase in incidence globally and a high mortality rate. Most CCAs are diagnosed in the advanced and metastatic phases of the disease, due to the paucity of signs and symptoms in the early stages. This fact, along with the poor results of the local and systemic therapies currently employed, is responsible for the poor outcome of CCA patients and strongly supports the need for novel therapeutic agents and strategies. In recent years, the introduction of next-generation sequencing technologies has opened new horizons for a better understanding of the genetic pathophysiology of CCA and, consequently, for the identification and evaluation of new treatments tailored to the molecular features or alterations progressively elucidated. In this review article, we describe the potential targets under investigation and the current molecular therapies employed in biliary tract cancers. In addition, we summarize the main drugs against CCA under evaluation in ongoing trials and describe the preliminary data coming from these pioneering studies.

## 1. Introduction

Cholangiocarcinoma (CCA) is the second most common primary liver cancer and accounts for about 3% of all gastrointestinal tumors [1]. CCA originates from the epithelium lining the biliary tree and can be divided into two main classes in accordance with the anatomical and cellular origin: intrahepatic CCA (iCCA) and extrahepatic CCA (eCCA) [2,3,4]. The latter can be further distinguished into perihilar CCA (pCCA) and distal CCA (dCCA). The anatomical boundary between iCCAs and pCCAs is formed by the second-order bile ducts, while the cystic duct is the anatomic limit between pCCAs and dCCAs. pCCAs represent the majority of tumors, accounting for 60–70% of them, while 20–30% are located distally, and intrahepatic CCA accounts for 5–10% [2]. This classification, other than anatomical, can be considered also “biological”, as each CCA subtype possesses unique biological and pathological properties, and thus, it can be targetable by distinct therapeutic options [5,6].

Besides liver flukes, which have been recognized as a causative agent for CCA, most of these tumors occur sporadically, without any apparent cause. Nevertheless, several risk factors for CCA have been identified, such as chronic biliary and liver diseases, and lifestyle-related aspects causing chronic inflammation and cholestasis [7]. The geographical distribution of known CCA risk factors is variable; this reflects the increased incidence rates of CCA observed in some areas of the world and explains the wide geographical differences in occurrence. For instance, the incidence rate is as high as 80 new cases per 100,000 inhabitants in Southeast Asia, whereas CCA occurrence ranges between 0.5 and 1.5 cases per 100,000 people in countries such as Canada [8]. Moreover, the incidence is increasing for iCCAs, in contrast to extrahepatic CCAs, which show rather a gradually decreasing trend [9,10].

CCA is an aggressive malignancy, with a median survival of less than 24 months from the time of the diagnosis [11]. This is mainly due to the fact that, at the time of diagnosis, only about 30% of the patients can be submitted to treatments with curative intent [12,13]. Consequently, the global five-year survival is still as low as 10% [14]. Surgery, the backbone of curative treatments for CCA, is effective in early, completely-resectable stages and in locally-advanced stages in combination with adjuvant chemotherapy (5-fluoruracil, gemcitabine, oxaliplatin, etc.) or adjuvant radiotherapy. Systemic therapies in unresectable and recurrent cases are associated with poor outcome. In addition, liver transplantation is not recommended for CCA because of the lack of clear selection criteria and the contrasting outcomes obtained [15,16], but the issue is still under investigation in further ongoing trials. Based on these epidemiological and clinical evidence, it is imperative that new therapeutic agents are developed for a significant improvement of the outcome of advanced CCA patients.

## 2. Potential Targetable Genes and Molecular Pathways

Early studies on the genetic pathophysiology of CCA focused, mainly due to technical limitations, on the elucidation of the role of single genes, often found mutated in other malignancies. These studies allowed demonstrating the role of *Kirsten rat sarcoma viral oncogene homolog* (*KRAS*), *tumor protein 53* (*TP53*), and *cyclin-dependent kinase inhibitor 2A* (*CDKN2A*) mutations in the onset and progression of CCA. Furthermore, genes affected by copy number alterations, like members of the ErbB receptor family, and their roles in the pathogenesis of CCA have been investigated [16].

In recent years, the advent of next-generation sequencing technology has substantially increased the investigative potential of scientists, while, at the same time, unravelling the extraordinary complexity of the molecular events occurring in biliary cancers, their interactions, and their relationships with risk factors and causative events. Among them, mutations in the genes of isocitrate dehydrogenases (*IDH1* and *2*) and fusions of *the fibroblast growth factor receptor 2* (*FGFR2*), as well as mutations in genes involved in chromatin-remodeling, like *AT-rich interaction domain 1A* (*ARID1A*), *protein poly-bromo1* (*PBRM1*), and *BRCA1-associated protein 1* (*BAP1*) (Table 1), were detected in iCCAs [17,18,19,20,21,22,23,24,25,26,27,28]. Of note, some of these altered genes can be either directly inhibited or the related pathways suppressed via specific inhibitors. In the following paragraphs, the potential targets of molecularly-driven therapies against CCA will be described.

### 2.1. Tyrosine Kinase Inhibitors

The tyrosine kinase signaling pathways include some of the most important membrane machineries for cell communication, and mutations of their components are often involved in human cancer. Activating mutations of the *epidermal growth factor receptor* (*EGFR*) gene are well-characterized in non-small cell lung cancer (NSCLC), breast, colorectal, head and neck cancer, and other malignancies [29,30]. These tumors are highly sensitive to tyrosine kinase inhibitors (TKIs), which have been recently adopted in clinical practice for the treatment of advanced-stage cases, representing a hallmark for genetically-tailored therapies for cancer. The tyrosine kinase family includes other members, including human epidermal growth factor receptor 2 (HER2/neu), vascular endothelial growth factor receptor (VEGFR), platelet-derived growth factor receptor (PDGFR), and fibroblast growth factor receptor (FGFR2), which can be altered by activating critical pathways in tumorigenesis, cancer progression, survival, resistance to chemotherapy, and metastasis [31]. Growing evidence about their involvement in the pathogenesis of CCA, and their use as targets for novel therapies has emerged in the last few years [21].

#### 2.1.1. ErbB Inhibitors

Epidermal growth factor receptor (EGFR, ErbB1, HER1) is a transmembrane protein of the ErbB tyrosine kinase receptors family, which includes also ErbB2 or HER2/neu, ErbB3, and ErbB4. EGFR is activated by binding to its specific ligands, including epidermal growth factor (EGF) and transforming growth factor α (TGFα). Upon activation, a transition from an inactive monomeric form of the receptor to an active homodimer or a heterodimer with another member of the same family (ErbB2/HER2/neu) takes place. Receptor dimerization stimulates its intrinsic intracellular protein-tyrosine kinase activity, autophosphorylation of several tyrosine residues in its C-terminal domain, and activation of several signal transduction cascades, including the RAS-RAF-MEK-ERK, PI3K/AKT/mTOR, and JAK/STAT pathways, which influence cell proliferation, differentiation, adhesion, and migration [18,32].

**Figure 1:**
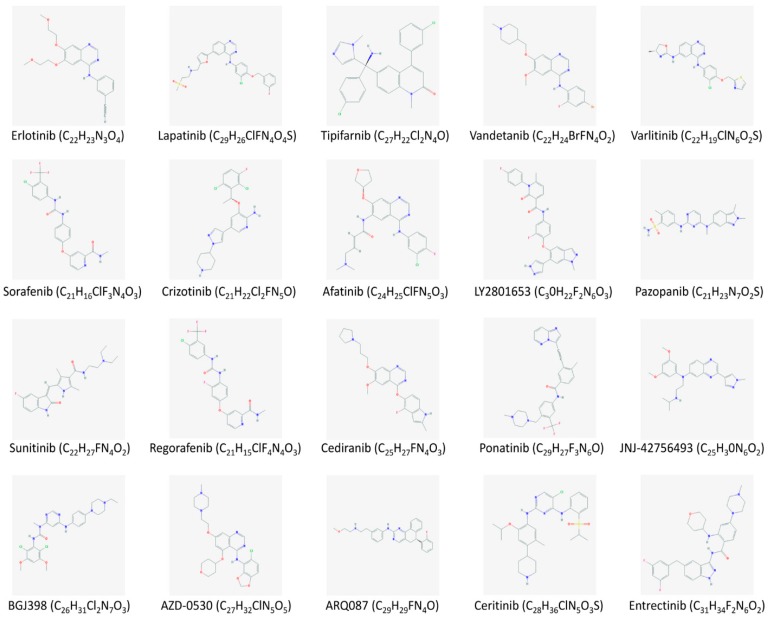
Chemical structures of the main tyrosine kinase inhibitors currently under investigation for targeted treatment of cholangiocarcinoma.

EGFR and HER2/neu overexpression has been detected in 10–32% of cases of iCCA [17,18,19]. Conversely, EGFR mutations are rare, occurring in only 11 of 492 (2.2%) iCCAs and 3 of 77 (3.8%) eCCAs [21]. Preclinical studies on the use of anti-EGFR and anti-HER2/neu inhibitors reported interesting findings [33]. Either some single arm phase II trials with erlotinib (EGFR tyrosine kinase inhibitor; Figure 1) or cetuximab (an anti-EGFR antibody) for the treatment of patients with advanced-stage disease showed encouraging results, which were further confirmed when the inhibitors were employed in combination with traditional chemotherapy (gemcitabine and oxaliplatin) [34,35]. Nevertheless, subsequent randomized phase II trials did not reveal any consistent advantage coming from the use of anti-ErbB therapies in association with chemotherapy, even when lapatinib (an EGFR and HER2/neu double inhibitor; Figure 1), panitumumab, or capecitabine were tested in various combinations [36,37,38,39,40]. A single randomized phase III trial was performed comparing erlotinib plus gemcitabine plus oxaliplatin with chemotherapy alone in advanced-stage biliary tract tumors. However, no significant differences were found in terms of median progression free survival (PFS) and median overall survival (OS) [41].

Javle et al. retrospectively evaluated five male patients with CCA presenting *HER2/neu* mutation or amplification who received anti-HER2/neu therapy with trastuzumab. The therapy was well-tolerated in all cases, but no oncological response was observed [42]. Trastuzumab in association with tipifarnib (a farnesyltransferase inhibitor of RAS kinase; Figure 1) is currently under investigation in an ongoing phase I trial (Table 2). Furthermore, Vandetanib, a promising multi-kinase inhibitor (Figure 1), has been tested alone and in combination with chemotherapy in phase I and II trials, but the improvement in terms of PFS and OS was negligible [43,44]. Additional multi-kinase inhibitors are under investigation in several phase I and II trials (Table 2).

#### 2.1.2. VEGFR and PDGFR Inhibitors

VEGFR is a family of receptors characterized by an extracellular domain for ligand binding, a transmembrane domain, and a cytoplasmic domain, including a tyrosine kinase domain. The molecular structure of VEGFR is similar to that of the PDGFR family members; however, the two receptor families have clear structural differences, like the number of extracellular immunoglobulin (Ig)-like domains (5 in PDGFR, 7 in VEGFR) and functional activities [45]. VEGF and its receptors are essential for angiogenesis, both in physiological conditions and various diseases, including cancer. VEGF was found to be overexpressed in 53.8% iCCAs and 59.2% extrahepatic CCAs, respectively, in a global cohort of 236 tumors; a statistically-significant association was found with intrahepatic metastases only in iCCAs [19,46]. In another study including 111 iCCAs, VEGFA was overexpressed in peripheral lesions, suggesting potential differences in pharmacological responses to anti-angiogenic therapies between the various types of CCA [47]. PDGF was found instead to be upregulated in CCA cell lines and 84.6% of human specimens in a study that showed also a positive correlation between PDGF expression and stage of the disease, metastatic dissemination, and poor prognosis [48].

Bevacizumab, a recombinant anti-VEGFA monoclonal antibody, has been tested in preclinical studies and demonstrated the ability to inhibit neoplastic vascularization and tumor growth both in vitro and in vivo [49]. In a phase II clinical trial, bevacizumab was tested in combination with gemcitabine and oxaliplatin in 35 patients with advanced disease; the median PFS was seven months, while the median overall survival was 12.7 months [50]. In addition, the combination of bevacizumab with erlotinib and FOLFIRI was investigated in other phase II trials, with the median OS ranging from 9.9–20 months [51,52]. A study investigating the association of bevacizumab with FOLFOX was closed for slow accrual, but a phase II clinical trial testing it in combination with gemcitabine and capecitabine is still ongoing (Table 2).

A series of multi-kinase inhibitors has been developed in recent years and tested in several neoplasms with promising results. The poor results of vandetanib were mentioned before; sorafenib (Figure 1), which acts also on PDGFR and Raf kinases, was employed alone in two phase II clinical trials with poor results [53,54]. In another double blind randomized phase II trial, sorafenib and gemcitabine were compared to placebo and gemcitabine; the PFS and OS were 4.9 and 3, and 11.2 and 8.4 months, respectively [55]. The combination with erlotinib was disappointing (mean OS six months), while that with gemcitabine – cisplatin was better (mean OS 14.4 months), both studied in phase II trials [56,57]. Ongoing studies with drug combinations including sorafenib are summarized in Table 2 Regorafenib (Figure 1), a promising agent sharing molecular features with sorafenib, is currently under investigation, as is ramucirumab (selective VEGFR2 inhibitor) and cediranib (VEGFR inhibitor; Figure 1). Nevertheless, the later showed poor results when added to gemcitabine and cisplatin [58]. Currently, there are no ongoing phase III clinical trials testing anti-angiogenic drugs in CCA, and this reflects the poor results described.

#### 2.1.3. FGFR Inhibitors

FGFR2 is one of the four members of the FGFR family of transmembrane receptors (a fifth member has been also identified, but it lacks the intracellular tyrosine kinase domain) [59]. These receptors are composed by an extracellular structure for the ligand composed of three immunoglobulin-like domains, a single transmembrane helix, and an intracellular domain with tyrosine kinase activity. The natural alternate splicing of the four FGFR genes results in the assembly of over 48 different receptor isoforms [60]. These isoforms differ in their ligand-binding and kinase domains, but all share the common extracellular region composed of three immunoglobulin (Ig)-like domains [61]. These isoforms interact with the largest family of growth factor ligands comprising 22 members, and as for other tyrosine kinase receptors, they homo- or hetero-dimerize in order to activate their kinase domain and activate intracellular cascades [62].

Genetic alterations in the *FGFR* genes, in particular fusions of *FGFR2*, have been described in 23% of 307 iCCAs and in none of the 36 extrahepatic CCAs [21]. This observation confirms the different pathophysiological features between intra- and extra-hepatic CCA and allows experimental testing of both selective and non-selective FGFR2 inhibitors, as well as monoclonal antibodies that target FGFR2. BGJ398 (Figure 1), a non-selective anti-FGFR inhibitor, increased cell death, and limited the neoplastic burden in mice and in a human-derived xenograft CCA model [63]. Ponatinib, another pan-FGFR inhibitor, has been employed with encouraging results in two patients with advanced disease who did not respond to chemotherapy. Currently, a series of ongoing trials are investigating the role of ponatinib (Figure 1), that of selective agents like BGJ398 and ARQ087 (Figure 1), and monoclonal antibodies (FRA144) in the treatment of patients with CCA and FGFR2 genetic alterations (Table 2).

#### 2.1.4. MET Inhibitors

Tyrosine kinase Met (c-MET) or hepatocyte growth factor receptor (HGFR), is encoded by the *MET* gene. It is a single-pass tyrosine kinase receptor essential for embryonic development, organogenesis, and wound healing. Hepatocyte growth factor/scatter factor (HGF/SF) and its splicing isoform (NK1, NK2) are the ligands of this receptor. When HGF/SF binds to the receptor, it induces its dimerization through a not yet completely understood mechanism, leading to its activation [64]. Abnormal MET activation is frequent in several cancers and has been found in 12–58% of iCCAs [27]. This finding led to the employment of MET inhibitors like cabozantinib (which inhibits also VEGF) in patients with advanced disease who had neoplastic progression after chemotherapy. Unfortunately, the results of this study were unsatisfactory (median PFS 1.7 months, median OS 5.2%) [65]. A better outcome was obtained in a phase I clinical trial with tivantinib, an oral MET inhibitor, and gemcitabine: among the 73 patients included, 20% and 46% had a partial and stable response, respectively [66]. Importantly, tivantinib was also better tolerated than cabozantinib. Currently, another MET inhibitor, LY2801653 (Figure 1), is under evaluation in a phase I clinical trial (Table 2).

#### 2.1.5. ROS1 (ALK) Inhibitors

ROS1 is a receptor tyrosine kinase (encoded by the *ROS1* gene) with an unknown physiological role, whose physiologic ligand has not yet been identified [67]. ROS1 has a structural similarity to the anaplastic lymphoma kinase (ALK) protein, and this makes it responsive to anti-ALK drugs such as crizotinib (Figure 1). Previously, crizotinib has been shown to be effective in the treatment of NSCLC patients with ALK mutations, and thus, it might be useful also for the treatment of CCAs [68]. Other ROS1 inhibitors, including ceritinib and entrectinib (Figure 1), are currently under investigation in phase II clinical trials including patients with CCA and ROS1 and/or ALK genetic alterations, which occur in 1.1–8.7% of the cases [27] (Table 2).

### 2.2. RAS/RAF/MEK/ERK Signaling Pathway Inhibitors

The RAS/RAF/MEK/ERK cascade comprises a series of cytoplasmic proteins that transport biological messages from the surface of the cell to the nucleus, especially the DNA, through the activity of MEK and ERK kinases. Oncogenic activation of this pathway is due to specific mutations into the kinase regions of the genes, producing a constitutive induction of the phosphorylating function of the RAS proteins, which in turn promotes neoplastic proliferation, differentiation, migration, and metastasis [69]. Mutations of KRAS, NRAS, BRAF, and other components of the cascade are well-known in several cancers, including gastrointestinal, pulmonary, and skin malignancies, and represent the substrate for the targeted therapies currently in use [69,70]. KRAS is mutated in 9.5% of iCCAs and 15.3% of extrahepatic CCAs, respectively, while respective figures for NRAS are 3.6% and 2.6%, according to Walter et al. [21].

**Figure 2:**
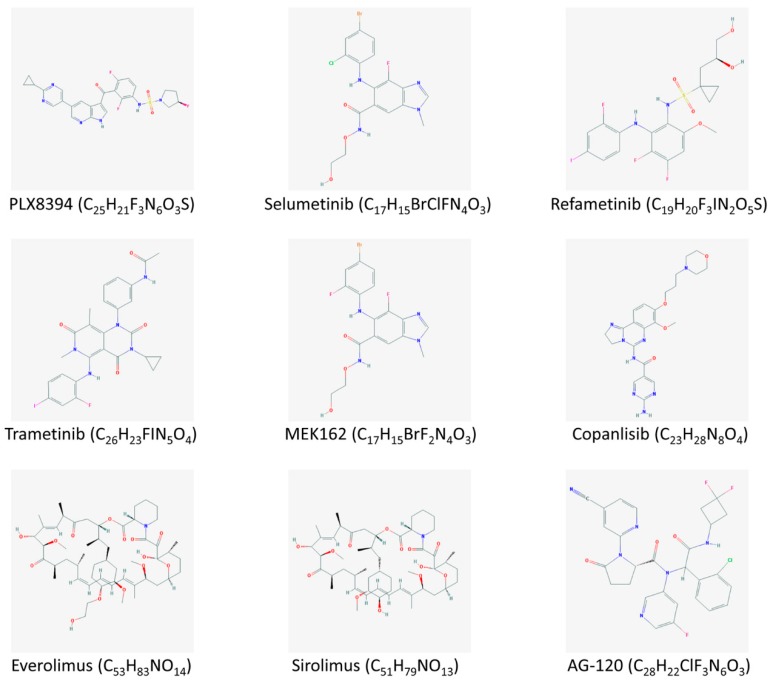
Chemical structures of promising agents included in ongoing clinical trials for targeted treatment of cholangiocarcinoma.

In contrast, BRAF was found mutated only in iCCAs (3.3%) [21]. BRAF has become an intriguing pharmacological candidate in recent studies, because of the failure to directly target mutant RAS in patients with cancer. Nevertheless, to date, only sporadic reports exist on the clinical use of vemurafenib, a specific inhibitor of BRAF V600 mutated kinase, in CCA patients. Silkin published a case with a complete clinical response after therapy with vemurafenib, panitumumab, and irinotecan in a patient who carried the V600 mutation [71]. Currently, an oral medication that appears to selectively bind to and inhibit the activity of both wild-type and mutated forms of BRAF, namely PLX8394 (Figure 2), is being employed in two phase I/II clinical trials with patients with advanced solid tumors including CCA (Table 2). Furthermore, regorafenib and sorafenib discussed earlier have the potential to inhibit BRAF [24].

MEK targeted inhibition appears to be promising for the treatment of advanced CCA. Selumetinib (Figure 2), an inhibitor of MEK1/2 proteins, has been evaluated alone or in combination with chemotherapy in small cohorts of patients in phase I and II clinical trials, with encouraging percentages of patients with stable disease (75% and 61%, respectively). An ongoing phase II trial is evaluating the combination of gemcitabine and selumetinib versus gemcitabine alone (Table 2). Furthermore, other promising MEK inhibitors, including refametinib, trametinib, and MEK162 (Figure 2), are being investigated in several clinical trials (Table 2). Trametinib alone has been investigated in 20 Japanese patients with several biliary cancers, with a 12-week stable disease rate of 10% and 2.7 months mean PFS [72].

### 2.3. PI3K/AKT/mTOR Signaling Pathway Inhibitors

PI3K/AKT/mTOR is an intracellular signaling cascade with essential roles in cell cycle regulation. PI3K activation phosphorylates AKT, which translocates to the plasma membrane and produces a series of downstream effects, including activation of mTOR, which in turn exerts some of the most relevant transcriptional functions of the pathway [73]. The cascade is physiologically inhibited by the *PTEN* tumor suppressor gene, but in numerous cancer types, it remains overactive, reducing apoptosis and allowing proliferation. *PI3K* mutations occur in 6.5% of extrahepatic CCA and 4.4% of iCCAs, whereas *PTEN* mutations have been detected in 4.4% and 3.9% of intra- and extra-hepatic CCAs, respectively [21].

Everolimus (Figure 2) is an mTOR inhibitor that is used for the treatment of several tumors, and it has been tested in combination with gemcitabine and cisplatin in patients with non-responsive to chemotherapy solid tumors, including 10 cases of biliary cancers (gallbladder and CCAs). Among them, six (60%) had stable disease. In another phase I trial, everolimus has been employed in combination with gemcitabine, but the results have not been published yet. Results from two further studies evaluating sirolimus (mTOR inhibitor; Figure 2) and copanlisib (PI3K inhibitor; Figure 2) are expected (Table 2). Another PI3K inhibitor, BKM120, has been used in combination with FOLFOX6 in a phase I trial including patients with solid tumors, resulting in high toxicity rates [74]. Finally, the use of MK2206 (which inhibits AKT) has been investigated in a recent phase II trial including eight cases of advanced biliary cancers; the authors achieved a PFS of 1.7 months and an OS of 3.5 months, without serious adverse events [75]. A study designed to combine the MEK1/2 inhibitor selumetinib with MK2206 in patients with unresectable biliary tract cancers was withdrawn prior to enrollment.

### 2.4. Glucose Metabolism Enzyme Inhibitors

The IDH enzyme catalyzes the oxidative decarboxylation of isocitrate, producing alpha-ketoglutarate (α-ketoglutarate) and CO_2_. Initially, the oxidation of isocitrate to oxalosuccinate takes place, followed by the decarboxylation of the carboxyl group beta to the ketone, forming alpha-ketoglutarate. The enzyme consists of three isoforms in humans: the IDH1 and IDH2 isoforms function outside the context of the citric acid cycle and localize to the cytosol, mitochondrion, and peroxisome, while IDH3 catalyzes the third step of the citric acid cycle in the mitochondria [76]. Mutations of IDH genes produce aberrant isoforms that convert isocitrate to the 2-hydroxyglutarate (2-HG) oncometabolite, which causes increased DNA methylation, cell proliferation, and angiogenesis [27]. IDH1 and IDH2 mutations have been found in gliomas and hematological malignancies, but also in iCCAs, with a respective incidence of 11% and 4.8% among 1094 cases reviewed [21].

Early results from one phase I clinical trial testing an IDH inhibitor are currently available. The study included 73 patientswith IDH1 mutated CCA who had been treated in the dose escalation (*n* = 24) and expansion (*n* = 49) cohorts with AG-120 (Figure 2). Among the 72 efficacy evaluable patients, 6% (*n* = 4) had a confirmed partial response and 56% (*n* = 40) experienced stable disease [77]. Further outcomes are expected, as well as from other trials employing AG-121 (IDH2 inhibitor) and AG-881 (pan-IDH inhibitor) (Table 2).

A phase II trial is currently recruiting patients to test rucaparib, another metabolic regulating anti-cancer drug, in combination with nivolumab (NCT03639935). Specifically, rucaparib is an inhibitor of the enzyme poly-ADP ribose polymerase (PARP), a protein involved in a number of cellular processes such as DNA repair, genomic stability, and programmed cell death.

### 2.5. Promising Targets and Inhibitors

The JAK/STAT signaling pathway brings information from extracellular biological signals to the nucleus, resulting in DNA transcription of genes involved in immunity, but also proliferation, differentiation, apoptosis, and oncogenesis. The JAK/STAT signaling cascade consists of three main components: a cell surface receptor, a Janus kinase (JAK1, JAK2, or TYK2) protein, and two signal transducer and activator of transcription (STAT) proteins [78]. Among the STAT proteins, STAT3 and STAT5 have been found to be the most frequently implicated in human cancers [79]. Dysregulated JAK/STAT activation has been detected in 50% of patients with iCCA (70% in iCCAs with an inflammatory background) [28]. Several agents able to inhibit components of the JAK/STAT pathway have been recently proposed for oncological therapies, and may represent interesting targets also in patients with CCA. Among the JAK inhibitors, the most attractive appears to be ruxolitinib (which inhibits both JAK1 and 2 proteins), as it has undergone several clinical studies and is approved for the treatment of myelofibrosis. Among the STAT3 inhibitors, the most relevant are OPB-31121, BBI-608, and BBI-503. The former showed encouraging results in a phase I clinical trial in patients with advanced hepatocellular carcinoma, while BBI-608 is currently evaluated in combination with traditional chemotherapy in several trials on gastrointestinal cancers (not CCA) [28,80]. Finally, BBI-503 is currently under evaluation in a phase II clinical trial, which recruits patients with advanced hepatobiliary cancer, and the results are anxiously expected (Table 2). Preclinical in vitro studies evidenced that BBI-608 downregulates the expression of β-catenin and c-Myc in pancreatic cancer cells [81].

The Wnt/β-catenin signaling pathway controls embryonic development and adult homeostasis, and is implicated in several cancers, including CCA. Thus, it may represent an attractive target for tailored therapies. Furthermore, the Notch signaling pathway, which has a central role in the determination of the biliary cell fate during development, as well as in chronic inflammation and carcinogenesis, can be a potential target. Indeed, Notch1 was found upregulated in CCAs, and preclinical studies confirm its role in cholangiocarcinogenesis. [20]. Currently, several Notch inhibitors are in development, such as γ-secretase inhibitors, blocking peptides, monoclonal antibodies, and decoys, which may represent potential candidates for clinical trials including CCA patients [82,83].

Somatic mutations in several chromatin-remodeling genes have been detected with different percentages in iCCA and extrahepatic CCA. In particular, *ARID1A*, *PBRM1*, and *BAP1* mutations were identified in 14%, 6.8%, and 8.8% of 501 iCCAs, respectively [21]. The corresponding figures for extrahepatic CCAs were 14.6%, 3.8%, and 1.5%. These findings suggest that alterations in chromatin remodeling may play a significant role in the pathogenesis of CCA and agents that act at this level, like vorinostat and panobinostat (histone deacetylase inhibitors), may harbor promising therapeutic potential.

The Hedgehog signaling pathway is a developmental pathway that transmits information required for proper cell differentiation to embryonic cells. It is implicated in several diseases, including cancer [84]. In a recent study, the expression levels of the Hedgehog signaling pathway components were evaluated in 50 surgical CCA specimens [85]. Among them, GLI1 was overexpressed in 24 (48%) and PTCH1 in 16 (32%) samples. Interestingly, more than 80% of cases with elevated PTCH1 also show a significant overexpression of GLI1, and SHH overexpression was found in seven tumors with PTCH1 or GLI1 upregulation [85]. In this study, Hedgehog signaling inhibition with BMS-833923 and gemcitabine reduced tumor volume in CCA xenografts. In another preclinical study, cyclopamine (another Hedgehog inhibitor) was able to attenuate the in vitro growth of CCA cell lines, as well as to restrain tumorigenesis in a CCA xenograft model [86]. Similar results were obtained with the use of vismodegib, an inhibitor of the non-canonical Hedgehog signaling, which induced growth and metastasis suppression in preclinical CCA models [87]. The suppression of the Hedgehog pathway in pancreatic cancer preclinical studies led to increased intratumoral vascularization and, thus, better intratumoral gemcitabine delivery, with transient stabilization of the disease [88]. This, in turn, brought clinical trials with Hedgehog inhibitors in pancreatic cancer. Such trials in CCA are expected, considering further evidence demonstrating that the Hedgehog pathway promotes desmoplastic response in CCA, which may limit the local distribution of chemotherapeutic drugs [89].

Another promising “druggable” target is represented by cancer-associated fibroblasts (CAF), which enrich the desmoplastic stroma of CCAs. In this context, CAFs produce several pro-oncogenic factors, like growth factors, chemokines, and matrix metalloproteinases, which induce neoplastic proliferation, tumor progression, and invasion [90]. Furthermore, these factors stimulate the creation of a fibrogenic peritumoral matrix, which precedes CCA onset and seems to function as a microenvironment promoting cancer development [91]. Agents with an inhibitory effect on this process, like 1D11 (a TGF-β inhibitor) and curcumin (a nutraceutical), inhibit the development of the fibrotic peritumoral support and, thus, the development and growth of CCA [92,93]. Currently, a clinical trial is planned to evaluate the safety and efficacy of curcumin in patients with primary sclerosing cholangitis (NCT02978339).

### 2.6. Rising Hopes from CCA Immunotherapy

Cumulative evidence indicates that the immune response also profoundly influences cholangiocarcinogenesis. Thus, it is not surprising that treatment able to modulate the immune systems is under intensive investigation in this disease [2]. In particular, immunotherapy is an emerging therapeutic approach based on the concept that supporting the immune system enhances its defenses and responses against cancer, thus improving the oncological outcome. Successful results have been obtained in recent years with the use of monoclonal antibodies targeting PD1/PD-L1 (nivolumab, pembrolizumab) and CTLA4 (ipilimumab) in the treatment of melanoma and NSCLC. In a study including 31 surgically-resected iCCAs and the corresponding healthy adjacent tissues, the authors detected higher expression rates of PD1 and PD-L1 in tumors [94]. Pembrolizumab has been tested in a phase I clinical trial including patients with biliary cancer PD-L1 positive; the response rate was 17%, and the medication was globally well tolerated [27]. Several ongoing trials are currently under evaluation for different immunotherapy agents in CCA (Table 2).

## 3. Conclusions

**Figure 3:**
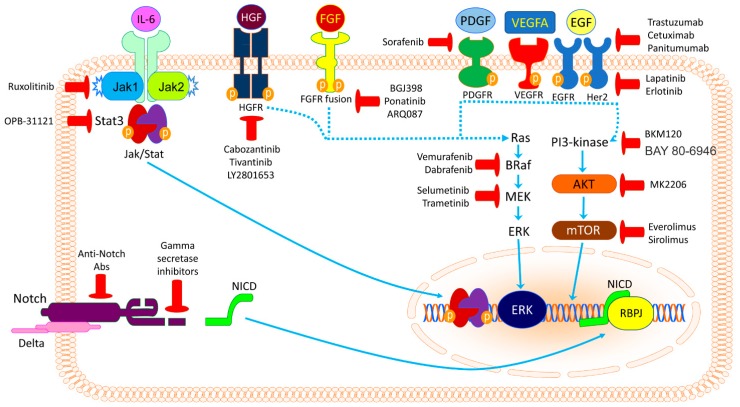
Overview of some of the molecular pathways deregulated in cholangiocarcinoma and the available inhibitors of these cascades.

Cholangiocarcinoma (CCA) is a highly fatal disease with a rising incidence worldwide. The lack of specific symptoms, as well as their late appearance results in the late diagnosis of the disease, when curative strategies cannot be successfully applied. In order to improve the prognosis of CCA patients significantly, a better understanding of the molecular pathogenesis of the disease is strongly required. In this regard, the advent of sophisticated methodologies able to provide researchers and clinicians with a comprehensive portrait of the mutational landscape of human CCA has triggered new efforts to identify novel therapeutic agents and strategies against this deadly disease. On the one hand, these modern approaches led, at least partly, to elucidating the pathophysiology of CCA and, consequently, the identification of potential candidates for molecularly-based therapies. On the other hand, the mounting body of information coming from these analyses has clearly shown that various human CCA subsets exist, characterized by heterogeneous molecular alterations. These alterations result in the activation of distinct signaling cascades, which should be selectively targeted and whose interplay is still poorly defined (Figure 3). Due to this molecular complexity, it is not surprising that the pioneering targeted approaches against CCA have produced unsatisfactory results to date. Additional investigations are needed to unravel the cellular effects of gene dysregulation (proliferation, apoptosis, migration, invasion, chemoresistance), the functional crosstalk between these molecular alterations, the biologic consequences (advantageous and detrimental) resulting from their inhibition, the compensatory pathways paradoxically activated by targeted therapies, and the eventual addiction to metabolic and hormonal stimuli deriving from such mutations. Furthermore, the interaction between CCA cells and the tumor microenvironment (CAFs, immune cells, non-parenchymal liver cells), a hallmark of cholangiocarcinogenesis, should be elucidated. The latter might allow the development of therapeutic strategies aimed at simultaneously targeting several cell types that are critically at play in CCA development and progression. For this purpose, ad hoc preclinical in vitro and in vivo models should be employed that faithfully recapitulate the molecular subtypes of the human disease. Finally, multiscale proteomic and metabolomic approaches should be applied both to identify novel druggable targets and to stratify CCA patients who might benefit from tailored therapies.

## Figures and Tables

**Table 1:** Incidence of mutations in targetable pathways in CCAs included into the COSMIC database. iCCA, intrahepatic cholangiocarcinoma; eCCA, extrahepatic CCA.

Gene	%	Comment
EGFR	1.6	2.2–20% in other series [17,18,19,20,21]. Overexpression of EGFR and/or HER2/neu has been documented in 10–32% of iCCAs [17,18,19].
VEGF	0.7	VEGF overexpression has been reported in about 54% of iCCAs [19].
KRAS	23	More common in eCCAs [20,21].
NRAS	4	Similar distribution between iCCAs and eCCAs [20,21].
BRAF	4	There were no BRAF mutations in 137 eCCA cases reviewed by Walter et al. They were detected in 3.3% of 723 iCCAs [20,21].
FGFR2	2.1	FGFR2 fusions were observed in approximately 3–50% of iCCAS [22,23,24].
MET	0.7	MET has been found overexpressed in 12–58% of iCCAs [25,26].
ROS1	0.7	In other reports, the frequency of ROS1 alterations varies between 1.1% and 8.8% [27].
PIK3CA	7	Slightly more frequent in eCCAs in accordance with Walter et al. [21].
PTEN	3.3	Similar distribution between iCCAs and eCCAs [20,21].
IDH1	9	Rare in eCCAs [20,21].
IDH2	3	Not found in eCCAs [20,21].
JAK1/2, STAT3	0.6–1%	JAK/STAT signaling pathway activated in 50–70% of iCCAS [28].
ARID1A	13	Similar distribution between iCCAs and eCCAs [20,21].
PBRM1	6	More common in iCCAs [20,21].
BAP1	9	Rare in eCCAs [20,21].

**Table 2:** Ongoing clinical trials for targeted therapies in advanced-stage cholangiocarcinomas.

Treatment	Target(s)	Phase	Identifier
Gemcitabine + oxaliplatin + capecitabine vs. gemcitabine + oxaliplatin + panitumumab + capecitabine	EGFR	II	NCT00779454
Gemcitabine + oxaliplatin + capecitabine + panitumumab or bevacizumab	EGFR − VEGFR	II	NCT01206049
Trastuzumab + tipifarnib	HER2/neu + FTI	I	NCT00005842
Varlitinib	EGFR	II	NCT02609958
Gemcitabine + oxaliplatin + cetuximab + trastuzumab + gefitinib + lapatinib + sorafenib + crizotinib	Multiple targets	I/II	NCT02836847
CART-EGFR	EGFR	I/II	NCT01869166
Afatinib + capecitabine	EGFR	I	NCT02451553
LY2801653 + cetuximab or cisplatin or gemcitabine or ramucirumab	Multiple targets	I	NCT01438554
Pazopanib + GSK1120212	Multiple targets	I	NCT01438554
Sunitinib	Multiple targets	II	NCT01718327
Gemcitabine + pazopanib	Multiple targets	II	NCT01855724
Regorafenib	Multiple targets	II	NCT02053376 NCT02162914
Gemcitabine + oxaliplatin + regorafenib	Multiple targets	II	NCT02386397
Ramucirumab	VEGFR	II	NCT02520141
Ramucirumab + pembrolizumab	Multiple targets	I	NCT02443324
Cediranib + AZD0530	Multiple targets	I	NCT00475956
Oxaliplatin + leucovorin calcium + fluorouracil + cediranib	Multiple targets	II	NCT01229111
Sorafenib	VEGFR − PDGFR − BRAF	II	NCT00238212
Sorafenib + oxaliplatin/capecitabine	VEGFR − PDGFR − BRAF	I/II	NCT00634751
Ponatinib	FGFR	II	NCT02265341 NCT02272998
JNJ-42756493	FGFR	I	NCT01703481
BGJ398	FGFR2	II	NCT02150967
ARQ087	FGFR2	II	NCT01752920
FRA144	FGFR2b	I	NCT02318329
Ceritinib	ROS1 − ALK	II	NCT02638909
Entrectinib	ROS1 − ALK	II	NCT02568267
LDK378 (Ceritinib)	ROS1 − ALK	II	NCT02374489
PLX8394	BRAF	I/II	NCT02428712 NCT02012231
Gemcitabine + selumetinib vs gemcitabine	MEK	II	NCT02151084
Refametinib	MEK	II	NCT02346032
Trametinib vs 5-fluoruracil or capecitabine	MEK	II	NCT02042443
Gemcitabine + MEK162	MEK	II	NCT01828034
Everolimus + gemcitabine	mTOR	I	NCT00949949
Sirolimus + gemcitabine	mTOR	I	NCT01888302
Copanlisib + gemcitabine	PI3K	II	NCT02631590
AG-881	IDH	I	NCT02481154
AG-120	IDH	I	NCT02073994
AG-120	IDH	III	NCT02989857
Rucaparib + nivolumab	PARP	II	NCT03639935
BBI-503	STAT3	II	NCT02232633
Pembrolizumab + GM − CSF	PD1	II	NCT02703714
Pembrolizumab	PD1	II	NCT02628067
Pembrolizumab	PD1	I/II	NCT02268825
Nivolumab or Ipilimumab	PD1 − CTL4	II	NCT02834013
